# Radiation Therapy in the Treatment of Head and Neck Rhabdomyosarcoma

**DOI:** 10.3390/cancers13143567

**Published:** 2021-07-16

**Authors:** Andrew J. Frankart, John C. Breneman, Luke E. Pater

**Affiliations:** Department of Radiation Oncology, University of Cincinnati, Cincinnati, OH 45267, USA; frankaaj@mail.uc.edu (A.J.F.); brenemjc@ucmail.uc.edu (J.C.B.)

**Keywords:** rhabdomyosarcoma, head and neck, pediatric cancer, radiation therapy

## Abstract

**Simple Summary:**

Rhabdomyosarcoma is a pediatric malignancy for which radiation therapy plays a significant role, along with surgery and chemotherapy. The approach to treatment varies by disease site as well as the histologic and molecular characterization of tumors. The head and neck represents a particularly complex location for radiation planning given the proximity of numerous critical structures. As a result, there is a great deal of complexity in terms of selecting radiation dose and technique that continues to evolve through large-scale trials by organizations such as the Children’s Oncology Group. This article aims to outline the management of head and neck rhabdomyosarcoma, describe the historical foundations of therapy for this disease, and provide a summary of current treatment recommendations, with a focus on radiation therapy.

**Abstract:**

The use of radiation therapy is an important part of multimodality treatment for rhabdomyosarcoma. The specific doses, treatment volumes, and techniques used in radiation therapy can vary dramatically based upon a number of factors including location, tumor size, and molecular characteristics, resulting in complex decisions in treatment planning. This article reviews the principles of evaluation and management for head and neck rhabdomyosarcoma including a summary of the historical studies upon which current management is based.

## 1. Introduction

Rhabdomyosarcoma (RMS) is a predominantly pediatric malignancy which can present throughout the body including the head and neck region. Given its radiosensitivity and the technical and cosmetic challenges of surgical resection in the head and neck, radiation has been a mainstay of treatment for tumors at this location. Unique challenges exist in light of the proximity of numerous critical organs-at-risk and the importance of minimizing late effects of treatment in the pediatric population. This review aims to outline key points in the management of head and neck rhabdomyosarcoma (HNRMS) and address considerations specific for radiation therapy at this disease site.

## 2. Results and Discussion

### 2.1. Background

Rhabdomyosarcoma is the most common pediatric soft tissue sarcoma [1]. Approximately 70% of patients present before age 10 with a peak incidence at ages three to five [2]. As this malignancy arises from embryonic primitive mesenchymal cells, it can manifest throughout the body in areas with skeletal muscle including the head and neck region in 40% of patients [3,4]. Most cases are sporadic, but RMS can be associated with Beckwith-Weidemann, Costello, and Li-Fraumeni syndromes [5,6,7]. Patients with abnormalities in the p53 pathway are more prone to aggressive anaplastic RMS with a high risk of secondary malignancy resulting from genotoxic chemotherapy and radiation [8].

**Figure 1:**
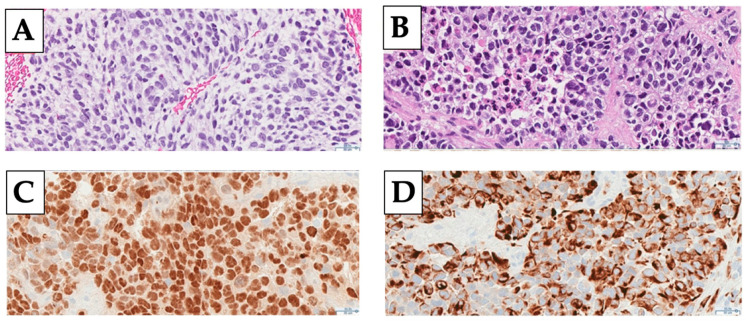
Representative images of the microscopic appearance of rhabdomyosarcoma including hematoxylin and eosin (H&E) staining of embryonal (Panel **A**) and alveolar (Panel **B**) subtypes and immunohistochemical stains for myogenin (Panel **C**) and desmin (Panel **D**). Images were obtained at 33.5× magnification.

The typical histological appearance of RMS is small round blue cells with high-grade features. Immunohistochemical (IHC) staining is classically positive for desmin and myogenin, reflecting the mesenchymal origin of RMS (Figure 1) [9]. Arrested myogenesis is a classic feature assessed using IHC. Historically, RMS has been characterized histologically into two main groups in the pediatric population: embryonal and alveolar. Embryonal histology is present in approximately 70% of pediatric cases and includes botryoid and spindle cell subtypes [10]. It tends to have a similar morphologic appearance to fetal skeletal muscle [11]. Approximately 20% of pediatric cases have been classified as alveolar histology which presents as undifferentiated small round blue cells in an alveolar or solid pattern [10,11]. Prognosis varies dramatically between these histologies with 5-year overall survivals of 82% for embryonal and 53% for alveolar HNRMS [11].

More recently, molecular characterization has replaced histology in classifying rhabdomyosarcoma. Fusion of PAX3 on chromosome 2 or PAX7 on chromosome 1 with FOXO1 on chromosome 13 is most commonly associated with alveolar histology. In approximately 20% of cases, patients with alveolar histology are fusion-negative and the natural history of their disease more closely resembles that of the favorable embryonal histology [12,13,14]. This has led to the adoption of the terms “fusion-positive” and “fusion-negative” to replace the historical terms of “alveolar” and “embryonal” [11]. Histological features have not been found to be predictive of fusion status, prompting increased inclusion of more advanced analytic techniques such as in situ hybridization (ISH) and polymerase chain reaction (PCR) in routine pathologic assessment of RMS [12,13]. Molecular classification is frequently being used in clinical practice and is required in current and future Children’s Oncology Group trials [15]. There is also active investigation into other FOXO1 fusion pathways to better delineate non-classical alveolar subtypes [16].

In addition to these factors, the anatomic location of rhabdomyosarcoma influences risk stratification and treatment approaches. Within the head and neck subsite, rhabdomyosarcoma tumor locations are grouped into three categories: orbital, parameningeal, and non-orbital/non-parameningeal [4]. Primary sites for RMS are broadly classified as unfavorable or favorable. Unfavorable sites include bladder, prostate, perineal/perianal, retroperitoneal, trunk, extremity, and parameningeal locations [2]. The parameningeal sites are the paranasal sinus, nasal cavity, nasopharynx, skull base, mastoid, middle ear, and infratemporal and pterygopalatine fossae [3]. As these parameningeal areas are located in the head and neck, it is particularly important to accurately assess tumor location in the diagnosis of HNRMS. Non-parameningeal head and neck sites including the orbit are considered favorable [2].

### 2.2. Diagnosis

The presenting symptoms of rhabdomyosarcoma are dependent upon the site of origin. Tumors arising in areas with space for soft tissue expansion can present as painless growing masses. Orbital tumors can result in proptosis, impairment of extraocular movement, visual disturbance, and pain. Parameningeal tumors can cause headaches as well as focal neurologic symptoms related to mass effect on adjacent normal structures. Cranial nerve palsies are frequent at presentation and can indicate skull base erosion and/or intracranial extension that could warrant expedited therapy [17]. HNRMS can often be initially mistaken for an infectious process, resulting in antibiotic administration and further evaluation after continued tumor growth.

Following concern on history and physical examination, formal diagnosis of HNRMS typically begins with cross-sectional imaging. Given the desire to avoid radiation exposure in children and the anatomic detail provided by magnetic resonance imaging (MRI), this imaging modality is preferred as an initial step in contrast to computed tomography (CT) imaging that would be used in a similar situation for an adult patient. Additionally, RMS tends to enhance similarly to normal muscle on CT and margins can be poorly defined [18]. CT can be helpful in delineating areas concerning for bone erosion given the negative prognostic significance and surgical implications of this finding and can also be used for dedicated evaluation of the chest for metastatic disease [19].

**Figure 2:**
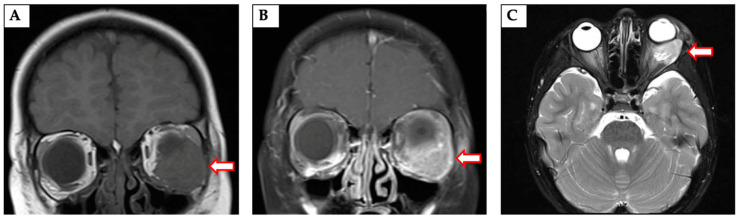
Magnetic resonance imaging (MRI) of an orbital primary tumor (arrows) in coronal T1 pre-contrast (Panel **A**) and post-contrast (Panel **B**) and axial T2 (Panel **C**) sequences. Panel (**B**) demonstrates the utility of contrast administration and fat suppression in delineating tumor for diagnosis and treatment planning.

The use of MRI with multiplanar reconstruction using routine spin-echo pre- and post-contrast T1 and T2 sequences can provide excellent resolution for tumor delineation [20]. HNRMS appears isointense to muscle on T1 sequences and hyperintense to muscle on T2 sequences (Figure 2) [18]. For orbital tumors, the addition of fat suppression with contrast administration and parallel alignment of the incline on sagittal series to the optic nerve can help improve tumor visualization [20]. Restricted diffusion is often seen on diffusion-weighted imaging and can have prognostic significance when viewed in conjunction with radiographic tumor metabolic activity [9,21]. Gradient echo sequences can be useful in assessing intracranial disease, but are prone to artifact generation at air-bone interfaces [20]. Given the prognostic importance of tumor location, it is important to carefully assess any parameningeal and intracranial extent of tumor on diagnostic imaging [22]. MRI can also be used for nodal staging, but benign enlargement of cervical and retropharyngeal lymph nodes is common in the pediatric population and this can lead to decreased reliability of size criteria for assessing risk of malignant involvement [20].

Biopsy using an open approach is preferred if possible [23]. It is important to obtain a sample adequate for both histologic and molecular classification. Additional tissue following biopsy is often not obtainable given the frequently limited surgical management of HNRMS. Once pathologic confirmation of the diagnosis is available, further staging imaging with PET is obtained with a focus upon regional nodal involvement and distant spread of tumor, though the sensitivity for nodal involvement is not optimal [24,25]. Use of PET for prognostication has not proven clinically valid [26]. Most patients also undergo a bone marrow biopsy along with cerebrospinal fluid (CSF) sampling via lumbar puncture if there is parameningeal disease [9]. An echocardiogram is typically performed to assess cardiac function prior to chemotherapy.

In light of limits for radiographic nodal staging for RMS and the presence of nodal disease in 9% of non-parameningeal and 18% of parameningeal cases, an additional consideration in the diagnostic evaluation is sentinel lymph node biopsy [27]. While more commonly utilized at other RMS disease sites, such as the extremities, use of sentinel node sampling was found to alter management in two of six patients with HNRMS in a single-institution case series including one patient with unexpected contralateral nodal disease [28]. This approach represents a less invasive strategy to accurately assess nodal involvement compared to neck dissection as used in squamous cell carcinoma in the adult population. Neck dissection is not utilized for staging in the HNRMS under normal circumstances.

### 2.3. Staging

The prognostic staging of rhabdomyosarcoma is commonly performed using parameters defined by the Intergroup Rhabdomyosarcoma Study Group (IRSG) and differs from most other tumor types. It utilizes a pre-treatment “Stage” that incorporates the primary tumor location, tumor size, and clinical nodal status and is prognostic of outcome as well as a post-surgery “Group” based upon the degree of resection completed prior to chemotherapy, which is useful in determining the role of radiotherapy for a patient (Table 1 and Table 2) [3]. Complete surgical resection is often not feasible for HNRMS in the pediatric population, given the risk for disfigurement and the technical challenges in resecting parameningeal disease, resulting in a Group III designation for many patients. Patients can be further classified into risk groups based upon stage, group, and fusion status. In terms of HNRMS, non-metastatic orbital and non-parameningeal head and neck cases are categorized as low-risk for fusion-negative tumors and intermediate-risk for fusion-positive tumors. Non-metastatic parameningeal tumors are categorized as intermediate-risk regardless of fusion status except in the rare circumstance when an R1 resection or better is achieved for fusion-negative disease [2].

### 2.4. Historical Rationale for Treatment Approach

#### 2.4.1. Intergroup Rhabdomyosarcoma Studies

Rhabdomyosarcoma has been treated in a multidisciplinary fashion by specialists in surgery and radiation oncology dating to the 1950s, with the addition of chemotherapy in the 1960s [29,30]. Early prospective investigations for the treatment of RMS were organized by the Intergroup Rhabdomyosarcoma Study Group (IRSG) in a series of four trials spanning from 1972 to 1997. IRS-I enrolled patients from 1972 to 1978 and assigned treatment regimens based upon post-surgery Group I to IV status as outlined previously. This study importantly demonstrated the ability to omit radiation in Group I patients with favorable histology, and included doses of radiation from 50 to 60 Gy delivered with supervoltage equipment. It also established the use of vincristine, actinomycin-D, and cyclophosphamide (VAC) chemotherapy in treating gross disease after surgical management. Overall survival at 5 years for all enrolled patients was 55%, but varied dramatically by group (83% for Group I vs. 52% for Group III) and primary site (89% for orbit vs. 55% for non-orbital/non-parameningeal head and neck, 47% for parameningeal) [31,32]. Overall survival continued to improve in subsequent IRS studies up to 71% at 5 years, and the benefit of using primary tumor location and staging in addition to group status to determine treatment was established. Standard radiotherapy doses of 50.4 Gy for gross disease and 41.4 Gy for microscopic disease were determined. The lack of benefit from hyperfractionated radiation and from alternative chemotherapy regimens apart from the standard of VAC for Group III disease was also shown. Importantly for HNRMS, the ability to use limited radiation volumes for parameningeal disease rather than whole brain radiation was demonstrated [10,33,34,35,36].

#### 2.4.2. Children’s Oncology Group

In 2000, the Children’s Oncology Group (COG) formed from the convergence of several independent investigative groups including the Intergroup Rhabdomyosarcoma Study Group (IRSG). The Soft Tissue Sarcoma Committee of the COG has driven subsequent research efforts in RMS [36]. For low-risk patients, the D9602 and ARST0331 studies were conducted to determine the safety of decreasing radiation and cyclophosphamide doses. D9602 showed that it was safe in the setting of favorable histology to reduce doses to 36 Gy for grossly resected Stage I primary tumors and to 45 Gy for Group III orbital disease, but that the complete elimination of cyclophosphamide might contribute to poorer outcomes, even in the lowest risk patients [37,38]. ARST0331 demonstrated the safety of these lower radiation doses as well as a shorter duration of chemotherapy with a reduced total cyclophosphamide dose (4.8 g/m^2^ vs. 28.6 g/m^2^ in D9602 and 26.4 g/m^2^ in IRS-IV) in patients with Stage I or II, Group I or II disease or Group III disease of the orbit [39]. However, the reduced orbital radiation dose of 45 Gy was insufficient if there was less than a complete response at week 12 [40]. Suboptimal control was noted for a slightly higher-risk group of patients with Stage I, Group III (non-orbit) or Stage III, Group I or II disease on the ARST0331 regimen [41].

For intermediate-risk patients, D9803 did not present a benefit to the addition of topotecan to VAC [42]. An analysis of local control outcomes on this study showed increased rates of local failure with tumor sizes of 5 cm or greater [43]. The use of intensity-modulated radiotherapy (IMRT) improved tumor coverage compared to three-dimensional conformal radiation therapy (3D-CRT) with no compromise in control with more conformal radiation fields [44]. ARST0531 attempted to improve local control using irinotecan and earlier radiation therapy at week 4, but this approach was inferior to that used in D9803 [45]. ARST1431 is an ongoing investigation evaluating the utility of temsirolimus in combination with VAC alternating with vincristine/irinotecan (NCT01222715). Temsirolimus shows activity against the mammalian target of rapamycin (mTOR) pathway, and was found to have utility in the setting of relapsed RMS [46]. This trial also utilizes an increased radiation dose of 59.4 Gy for tumors 5 cm or larger, given their association with increased local failure rate [45]. Additionally, ARST1431 gives the option for delayed primary excision when gross total resection is safe after induction chemotherapy, which may decrease the necessary radiotherapy dose.

### 2.5. Current Approach to Treatment in the Head and Neck

Based upon the findings of the aforementioned foundational studies, the general treatment paradigm for non-metastatic HNRMS includes maximal safe surgery (often biopsy only for HNRMS) followed by multiagent chemotherapy and radiotherapy, and then additional chemotherapy.

#### 2.5.1. Surgery

For RMS as a whole, upfront surgical resection is preferred and directly influences risk stratification per the Group I to IV classifications previously described. In some cases, gross total resection (GTR) with negative margins (i.e., R0 resection) can result in the avoidance of radiotherapy, which is a preferable situation in many cases given the acute and late toxicities of radiation in children. In the head and neck, an R0 resection is usually not feasible, particularly in technically challenging cases such as orbital and parameningeal tumors. Given the inability to avoid radiation in the absence of Group I status and the potential for cosmetic and functional impacts from surgery, aggressive upfront surgical management for HNRMS is generally not recommended. The Ablative surgery, MOld technique with afterloading brachytherapy, and immediate surgical Reconstruction (AMORE) approach has been used in carefully selected cases of advanced and recurrent HNRMS with acceptable outcomes including toxicity [4]. If attempted, surgical resection in HNRMS should ideally be performed by individuals specializing in head and neck surgery who have experience with reconstructive techniques. Sentinel lymph node assessment is recommended for staging over neck dissection, and grossly involved nodal disease should be excised if possible.

The use of delayed primary excision (DPE) following tumor response with chemotherapy represents an active avenue of investigation and has been used at other RMS disease sites to reduce radiation exposure [47]. This approach is optional on the current ARST1431 protocol, as it was on the D9803 trial if resection will not compromise form or function [42,45]. However, HNRMS poses a unique oncologic and reconstructive challenge with surgery, so DPE might not be as easily adapted at this site.

#### 2.5.2. Chemotherapy

The doses and schedules of chemotherapy for low- and intermediate-risk disease are currently driven by the most recent COG protocols for each group. In either case, vincristine, actinomycin-D, and cyclophosphamide (VAC) form the backbone of therapy. For low-risk patients, this can be alternated with vincristine/actinomycin-D (VA) alone in order to decrease a patient’s exposure to alkylating chemotherapy agents. For intermediate-risk disease treated on ARST1431, VAC is alternated with vincristine/irinotecan (VI) with or without temsirolimus (NCT01222715). VAC alone can also be utilized as per D9803 for patients who are not on trial. Chemotherapy is typically given concurrently with radiation with the omission of actinomycin-D to avoid excess toxicity.

An ongoing topic of debate is the necessary dose of cyclophosphamide. As outlined above, there have been consistent efforts to reduce the cyclophosphamide dose to preserve fertility and avoid late complications such as myelodysplasia and secondary leukemias [39]. This has resulted in decreased doses per cycle of cyclophosphamide from 2.2 g/m^2^ on IRS and early COG studies to 1.2 g/m^2^ on more recent COG trials [42]. Based upon this change in per-cycle dosing, as well as a reduction in the length of therapy, the cumulative doses of cyclophosphamide have also decreased. For example, this dose was 28.6 g/m^2^ on D9602 and decreased to 4.8 g/m^2^ on ARST 0331 in the low-risk group [37,39]. While this resulted in adequate control for lower risk subsets of patients, there is concern that excessive reduction in cyclophosphamide could compromise local control. A similar decrease was seen in the intermediate-risk setting between D9803 and ARST0531 [42,45]. Institutional data for the specific subsite of HNRMS show an association between cyclophosphamide doses greater than 20 g/m^2^ and improved local control [48]. It is not yet clear whether the total dose of cyclophosphamide or the pattern in which it is delivered most impacts outcomes [45]. As a result, the optimal balance between toxicity and benefit for cyclophosphamide remains an area of investigation.

#### 2.5.3. Radiation Therapy

Radiation therapy is indicated for all patients apart from completely resected fusion-negative tumors and therefore is almost always utilized in HNRMS. Intermediate-risk patients, including those with fusion-positive tumors, always receive radiation therapy regardless of the degree of resection [2]. Radiation is delivered in 1.8 Gy per fraction and is typically given after week 12 of chemotherapy with concurrent vincristine alone for low-risk disease and vincristine and cyclophosphamide for intermediate-risk disease. Radiation may be started earlier if a patient is symptomatic from their tumor or has intracranial extension, but these tumors are often very responsive to chemotherapy. As a result, radiotherapy can be delayed to week 13 for most patients [49].

Doses for primary tumors include 36 Gy for microscopic residual disease and resected unfavorable histology disease and 50.4 Gy for gross tumor. Group III disease of the orbit could previously be treated to 45 Gy with a complete response to chemotherapy at week 12, but this approach is no longer encouraged by the COG [40]. The current ARST1431 protocol allows for dose escalation to 59.4 Gy for tumors 5 cm or more in size, based upon the results of D9803 [43]. Involved nodal levels receive 41.4 Gy if nodal disease was resected and 50.4 Gy if gross disease remains, but elective radiation is not given to uninvolved nodal regions [2]. However, for patients not enrolled on protocol, some clinicians do give elective neck radiation to patients with fusion-positive tumors due to the higher risk of nodal failure in this population.

Initial tumor volumes should encompass all gross disease at diagnosis with a 0.5 to 1 cm expansion for microscopic disease as a clinical target volume (CTV). A decreased volume including only residual disease can be considered after 36 Gy for patients whose tumors responded to chemotherapy prior to radiation [50]. For disease in locations prone to changes in the volume of tumor and adjacent soft tissue, it is important to use image guidance during treatment to identify when adaptive planning is needed [51]. Metastatic sites can be managed with site-directed radiotherapy, though chemotherapy represents the foundation for treatment of this situation.

### 2.6. Overview of Techniques for Radiation Therapy

**Figure 3:**
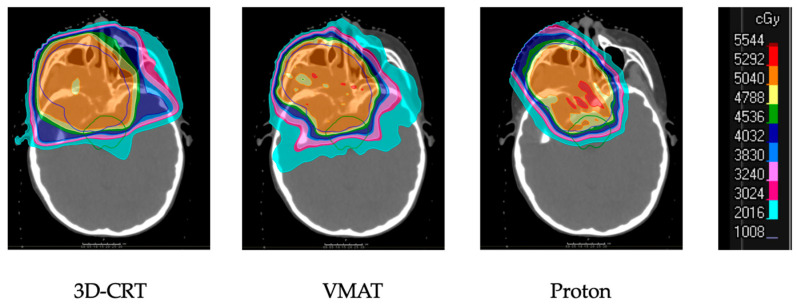
Comparison of plans for a parameningeal primary tumor using three-dimensional conformal radiation therapy (3D-CRT), volumetric arc-based therapy (VMAT), and proton therapy, showing improved dose conformality and sparing of normal tissue with proton therapy. Scale on the right shows dose in centigray (cGy).

Across all disease sites, the techniques of radiotherapy delivery have changed dramatically over the past several decades and RMS is no exception to this trend. In the early years of treatment, radiation was delivered using two-dimensional planning based upon plain radiographs, resulting in less precise delivery of radiation dose. With the advent of CT imaging, a transition to three-dimensional planning occurred, which has continued to the present day with expansions beyond 3D-CRT to modalities which provide even greater dose conformality including IMRT and proton therapy (Figure 3).

The 3D-CRT technique uses multiple fields of pre-determined shape and intensity to deliver dose to a target volume delineated on CT imaging. IMRT and the related approach of volumetric-modulated arc therapy (VMAT) allow for dynamic shaping of the beam using collimation with tungsten leaflets and can be given in static or rotational forms to increase the conformality of dose with a goal of reducing toxicity as a result [52]. This provides an advantage of reduced dose to adjacent normal structures, but requires more careful delineation of target structures since there is less prescription dose to target margins as compared to 3D-CRT. Outcomes with IMRT were compared to those with 3D-CRT on an analysis of the intermediate-risk D9803 trial that showed no differences in locoregional control or failure-free survival; target coverage was improved with IMRT on dosimetric analysis [44].

**Figure 4:**
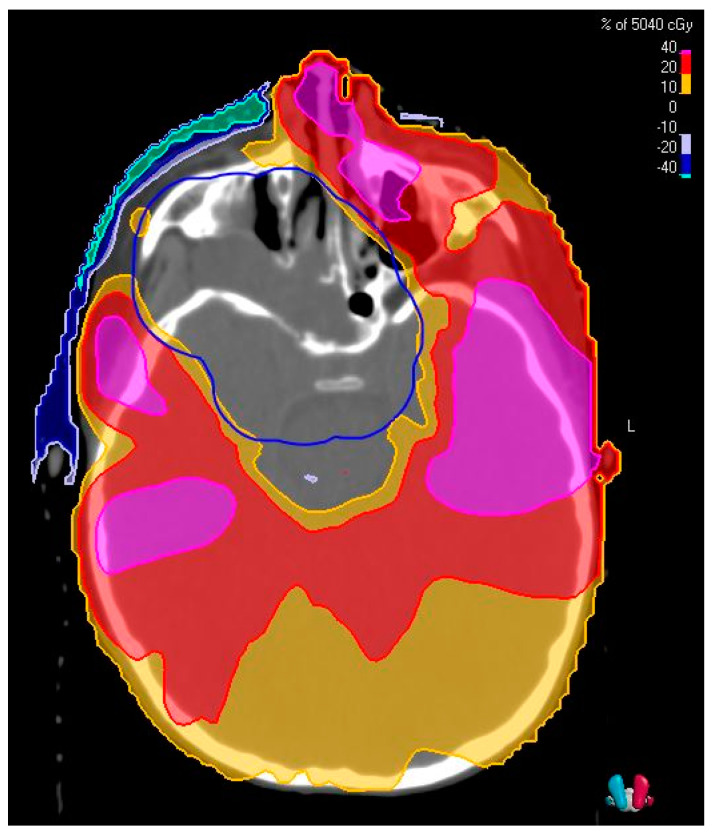
Difference in relative dose of a treatment plan as percentage of 5040 cGy when using volumetric arc-based therapy (VMAT) compared to proton therapy. Positive numbers on the scale indicate increased dose with VMAT.

Proton therapy has become more broadly available and utilized. This is commonly used in the treatment of pediatric cancers due to its ability to further reduce dose to adjacent normal tissue (Figure 4). It is also able to minimize the overall dose to the patient, compared to IMRT, and this has implications on the risk of secondary malignancy [53,54]. In contrast with conventional photon-based treatment, proton therapy utilizes relatively heavy, positively charged particles to deliver dose to a specified depth with little to no exit dose. Proton therapy can be delivered in several ways, including passive scatter and pencil beam scanning. Passive scatter uses physical devices at the machine head to distribute dose, while pencil beam scanning employs magnets for dose-painting on the target on a layer-by-layer basis. Both approaches have demonstrated clinical efficacy, though the use of pencil beam scanning often results in decreased total body dose and increased normal tissue avoidance compared to passive scatter treatment [55].

Within the broader category of pencil beam scanning proton therapy, there are two main approaches to treatment planning relevant to disease in the head and neck. Single-field optimization (SFO) utilizes uniform coverage of a target by multiple beams, while multi-field optimization (MFO) modulates dose on a per-beam basis. When treating volumes with irregular shapes or adjacent to critical structures, the use of MFO planning can create sharper dose distributions and is often used in HNRMS for parameningeal tumors. However, the higher degree of dose modulation for each individual beam can make these plans more susceptible to interfractional changes such as increased edema during treatment due to the impact of changing tissue density on proton dose deposition. As a result, MFO plans require careful monitoring using daily cone-beam CT imaging over the course of treatment with plan adjustments as needed to account for anatomical changes. Adaptive therapy to address such differences on a per-fraction basis is an area of ongoing investigation.

Brachytherapy can also be used in select cases. This most often involves placement of catheters immediately adjacent to or within the target volume and subsequent loading of a radioactive source to deliver dose. A brachytherapy approach can result in a favorable dose distribution and allow for dose escalation in some cases, but catheter placement is technically challenging in the head and neck region and outcomes are user-dependent. As a result, this approach should only be used in limited situations and by those with extensive brachytherapy expertise.

### 2.7. Considerations after Radiation Therapy

Per the most recent COG trial, follow-up includes a physical exam and imaging of the primary site and adjacent nodal basins every 3 months for the first year after treatment. These assessments can then be spaced to every 4 months in years 2 and 3 post-treatment and to every 6 months in year 4 (NCT01222715). Relapse tends to occur within the first 3 years following treatment and presents at the original primary site in 75% of cases with a typically poor prognosis [9]. Intracranial relapse is a less common event, but can be more frequently seen in HNRMS with intracranial extension or cranial nerve palsy at presentation and is associated with a particularly short expected survival after recurrence. Most patients with intracranial relapse are palliated with radiation therapy and salvage chemotherapy. CSF-focused I-131-labeled monoclonal antibody therapy has also been explored [56].

A number of specific post-treatment late effects also require close monitoring and management in the years after oncologic therapy is completed. Dental abnormalities are often seen following treatment for HNRMS. Both chemotherapy and radiation are felt to contribute. Frequently observed sequelae include dental root shortening, partial and total adontia, enamel damage, and delayed eruption [57,58]. Xerostomia from irradiation of the salivary glands can lead to multiple effects including dysgeusia, dysphagia, and an increased risk of dental caries.

Facial asymmetry is also commonly observed in follow-up after radiotherapy given the young age of a typical RMS patient, and can manifest as reduced facial depth and height and shortened mandibular length [57,59]. These changes remain a concern when using modern techniques such as IMRT, with up to 77% of patients having facial disfigurement and 10% of patients requiring cosmetic surgery in one series with a median of 7.7 years of follow up [58,60]. Changes in facial anatomy can lead to impacts on self-image in the future with one study showing over 50% of evaluated HNRMS survivors negatively rating their facial appearance in multiple domains at a median follow-up of 11.5 years [61]. Disfigurement can also occur due to lymphedema, particularly in cases where surgical or radiotherapeutic treatment of cervical adenopathy is required.

Ophthalmologic sequelae can happen after treatment for periorbital tumors. While conservation of the globe can typically be achieved, the majority of patients in a case series spanning from 1975 to 2010 reported some degree of ophthalmologic dysfunction including changes in visual acuity. Damage to the palpebral and ocular surfaces can also occur. Cataract formation is common after exposure of the lens to radiation, but is often reversible with surgical repair [62,63]. The use of proton therapy can aid in limiting the risk of bilateral cataract formation [64]. Hearing can also be impacted, depending on the tumor location and the proximity of critical structures such as the cochleae.

Pituitary exposure to radiation can result in deficiencies in multiple hormones, often in a dose-dependent fashion [65]. Growth hormone (GH) is often first affected and rates of GH deficiency have been reported as 50 to 100% following doses of 30 to 50 Gy [66]. This is especially important given the young age at presentation for many RMS patients and the proximity of the pituitary gland and hypothalamus to several parameningeal sites and the posterior orbit. Thyroid stimulating hormone (TSH) and adrenocorticotropic hormone can be affected at similar radiation doses at rates of 3 to 6%, though higher rates of TSH deficiency up to 24.5% have been reported for doses of 20 to 40 Gy [65]. Gonadotropin levels can also be detrimentally affected by radiation and affect pubertal development as well as compound the detrimental impact of cyclophosphamide on fertility [66]. With the use of proton therapy, doses to the pituitary can be decreased in many cases. This might lead to a reduction in risk of endocrinopathy given the dose-response relationship of this complication with radiation exposure.

Psychomotor development represents an additional major consideration with radiation treatment involving intracranial fields. This is of particular concern in younger patients at earlier stages of neurocognitive development. The impact on cognitive function appears to be dose-dependent based upon experiences with other intracranial disease sites [67]. Therefore, the use of proton therapy may reduce the impact on cognition and development. The PENTEC initiative has published modeling of this risk and continues to refine risk predictions for neurocognitive and other late effects of radiation [68].

Finally, secondary malignancy is a rare, but serious, long-term risk of radiation therapy. This risk is felt to relate to the total integral dose to the body [54]. Historical data of patients treated from 1973 to 2000 showed a 1.7% risk for developing a cancer after treatment for rhabdomyosarcoma with a higher risk with combined modality approaches [69]. Secondary malignancy rates of up to 6.7% reported at a median follow up of 7.7 years following IMRT for HNRMS have been reported, though the cases in this series were hematologic cancers and included only 30 patients from a single institution [60]. Further follow-up is needed to determine the risk of secondary malignancy associated with proton therapy, but it is theorized to be lower, based upon decreased integral dose to patients.

## 3. Materials and Methods

References for this article were compiled using an electronic search of the PubMed database using the search term “rhabdomyosarcoma” to identify articles published since 2000 with an emphasis on original manuscripts with multi-institutional results when available; when insufficient results to address a specific topic were found, a supplementary term was added to the “rhabdomyosarcoma” term to expand the search on that topic (e.g., “radiology”, “proton therapy”). Following this initial search strategy, the database was queried using the names of the foundational studies by the Children’s Oncology Group and its predecessors outlined in Section 2.4 to provide historical context for the treatment of rhabdomyosarcoma.

## 4. Conclusions

Head and neck rhabdomyosarcoma is a complex condition with a variable prognosis based upon the subsite of presentation and the histologic and molecular subtype. It requires multidisciplinary collaboration between surgical, medical oncology, and radiation oncology subspecialties for treatment as well as other subspecialties for medical and toxicity management. The techniques available for radiation therapy have progressed dramatically in recent decades and proton therapy is now generally recommended when available. The close proximity of numerous adjacent normal structures leads to the potential for a range of late toxicities which require monitoring in the years following the completion of therapy.

## Figures and Tables

**Table 1:** Staging per Intergroup Rhabdomyosarcoma Study Group (IRSG) classifications.

Stage	Site	Tumor Size	Nodal Involvement	Metastatic Spread
I	Favorable	Any	Any	No
II	Unfavorable	≤5 cm	No	No
III	Unfavorable	≤5 cm >5 cm	Yes Any	No No
IV	Any	Any	Any	Yes

**Table 2:** Grouping per Intergroup Rhabdomyosarcoma Study Group (IRSG) classifications.

IRSG Grouping	Description
**Group I**	**Localized disease with microscopic margin-negative (R0) resection** A*–*Confined to muscle/organ of presentation B*–*Extension beyond muscle/organ of presentation
**Group II**	**Gross total resection** A*–*Margin-positive resection (R1) B*–*Regional lymph node involvement with R0 resection of nodal disease C*–*Regional lymph node involvement with R1 resection of nodal disease
**Group III**	**Gross residual disease following resection** A*–*Biopsy only B*–*>50% resection completed
**Group IV**	**Distant metastatic disease at presentation**
